# Supplementary material for: A root cap-localized NAC transcription factor controls root halotropic response to salt stress in Arabidopsis
Source: Nat Commun. 2024 Mar 7;15:2061. doi: 10.1038/s41467-024-46482-7 (PMC10917740; doi:10.1038/s41467-024-46482-7)
Supplement: Supplementary file 1 — Supplementary Information [file 41467_2024_46482_MOESM1_ESM.pdf]

Supplementary information

A root cap-localized NAC transcription factor controls root halotropic response to salt stress in Arabidopsis

Lulu Zheng[1,2,3], Yongfeng Hu[4], Tianzhao Yang[5], Daoyuan Wang[1], Zhen Wang[1], Letian Jia[1], Yuanming Xie[1], Long Luo[5], Weicong Qi[6], Yuanda Lv[6], Tom Beeckman[2,3], Wei Xuan[1]*, Yi Han[5]*

[1]Sanya Institute of Nanjing Agricultural University, State Key Laboratory of Crop Genetics & Germplasm Enhancement, College of Resources and Environmental Sciences, Nanjing Agricultural University, Nanjing, China

[2]Department of Plant Biotechnology and Bioinformatics, Ghent University, Technologiepark 71, Ghent B-9052, Belgium

[3]VIB-UGent Center for Plant Systems Biology, Technologiepark 71, Ghent B-9052, Belgium

[4]Key Laboratory of Three Gorges Regional Plant Genetics and Germplasm Enhancement, Biotechnology Research Center, China Three Gorges University, Yichang, China.

[5]National Engineering Laboratory of Crop Stress Resistence Breeding, School of Life Sciences, Anhui Agricultural University, 230036 Hefei, China

[6]Excellence and Innovation Center, Jiangsu Academy of Agricultural Sciences, Nanjing 210014, China

This PDF file includes:

Supplementary Figure 1 to 22

Supplementary Table 1

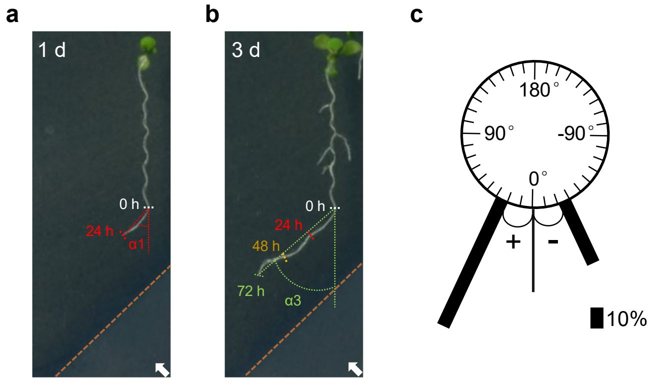

Supplementary Fig. 1. Quantification of root halotropic curvature. Related to Fig. 1.

a, b Representative images of root halotropic growth for 1 day (**a**) and 3 days (**b**) following halo-stimulation. a straight line was drawn connecting the location of the root tip at the indicated time points and at the zero time point during halo-stimulation, and the angle of the root bending from the vertical (set to 0°) was measured. α1 and α3 represent the bending angles at 1 day (**a**) and 3 days (**b**) of halo-stimulation, respectively. **c** Diagram showing the angles of the root tip response to salinity at the indicated time points following halo-stimulation. Percentages of roots in angle categories of 10 degrees, with the number (n) of roots measured, were quantified. Vertical angle sets to 0°. The angle away or toward from the salt gradient is defined as a positive (+) or negative value (-), respectively. The length of the bars represents the relative number of roots per category.

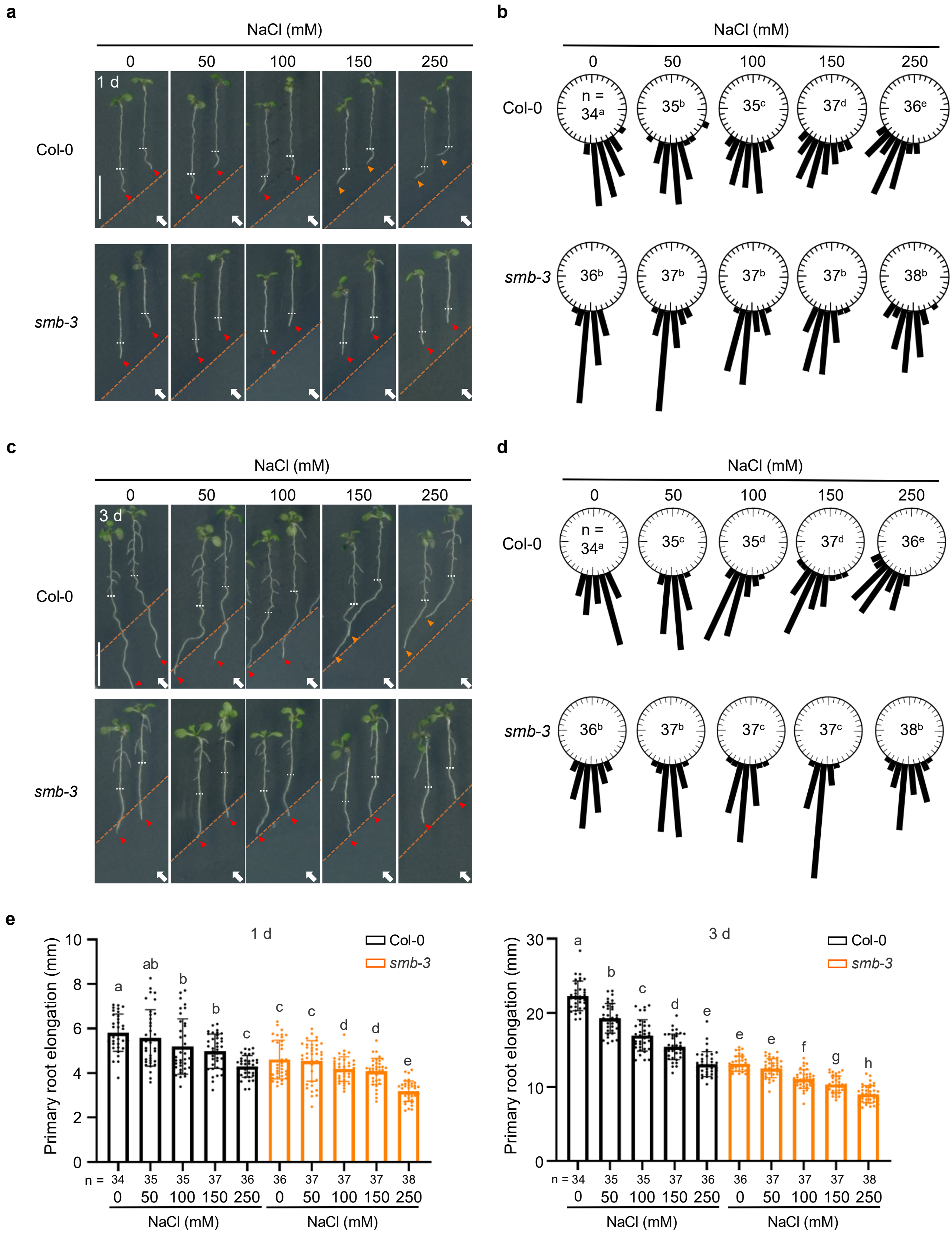

Supplementary Fig. 2. Comparison of sensitivity between wild-type and *SMB* knock-out mutants to different salt gradients. Related to Fig. 1.

a–**d** Halotropic root response of Col-0 and *smb-3* seedlings that were transferred to split-agar medium containing different concentrations of NaCl for 1 day (**a**) and 3 days (**c**). White arrows indicate the direction of NaCl diffusion; white dotted lines represent the initial location of the root tip when the salt gradient was created; orange dotted line represents mock-mock or mock-salt boundary; orange arrows represent the occurrence of halotropic root bending; while red arrows indicate the absence of halotropic root bending. Scale bar, 1 cm. Halotropic root curvature was quantified at 1 day (**b**) and 3 days (**d**). **e** Quantification of primary root elongation in Col-0 and *smb-3* seedlings that were transferred to split-agar medium containing different concentrations of NaCl for 1 day and 3 days. Values are means ± SD. In **b**, **d** and **e**, n indicates the number of independent seedlings, and alphabets denote significant differences ($P < 0.05$, two-way ANOVA by Tukey's test).

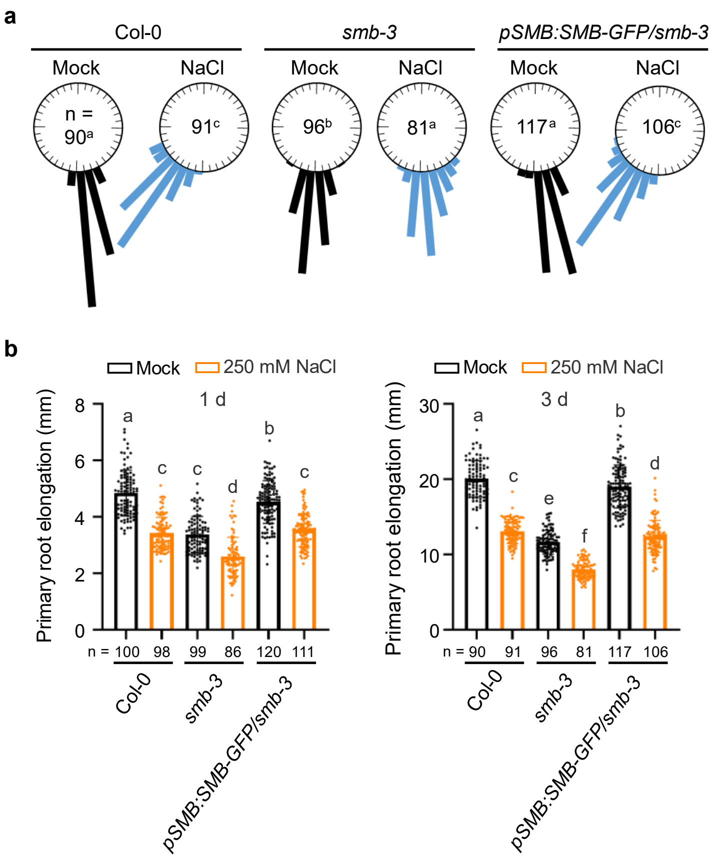

Supplementary Fig. 3. The effects of SMB on root halotropic bending and root elongation. Related to Fig. 1.

a Halotropic root curvatures of Col-0, *smb-3*, and its complemented line seedlings that were transferred to split-agar medium with or without 250 mM NaCl for 3 days. The angles were grouped on the wheel diagram as percentage of root bending. Black and blue bars represent the Mock and NaCl treatments, respectively. **b** Quantification of primary root elongation in Col-0, *smb-3*, and its complemented line seedlings that were transferred to split-agar medium with or without 250 mM NaCl for 1 day and 3 days. Values are means ± SD. In **a** and **b**, n indicates the number of independent seedlings, and alphabets indicate significant differences ($P < 0.05$, two-way ANOVA by Tukey's test).

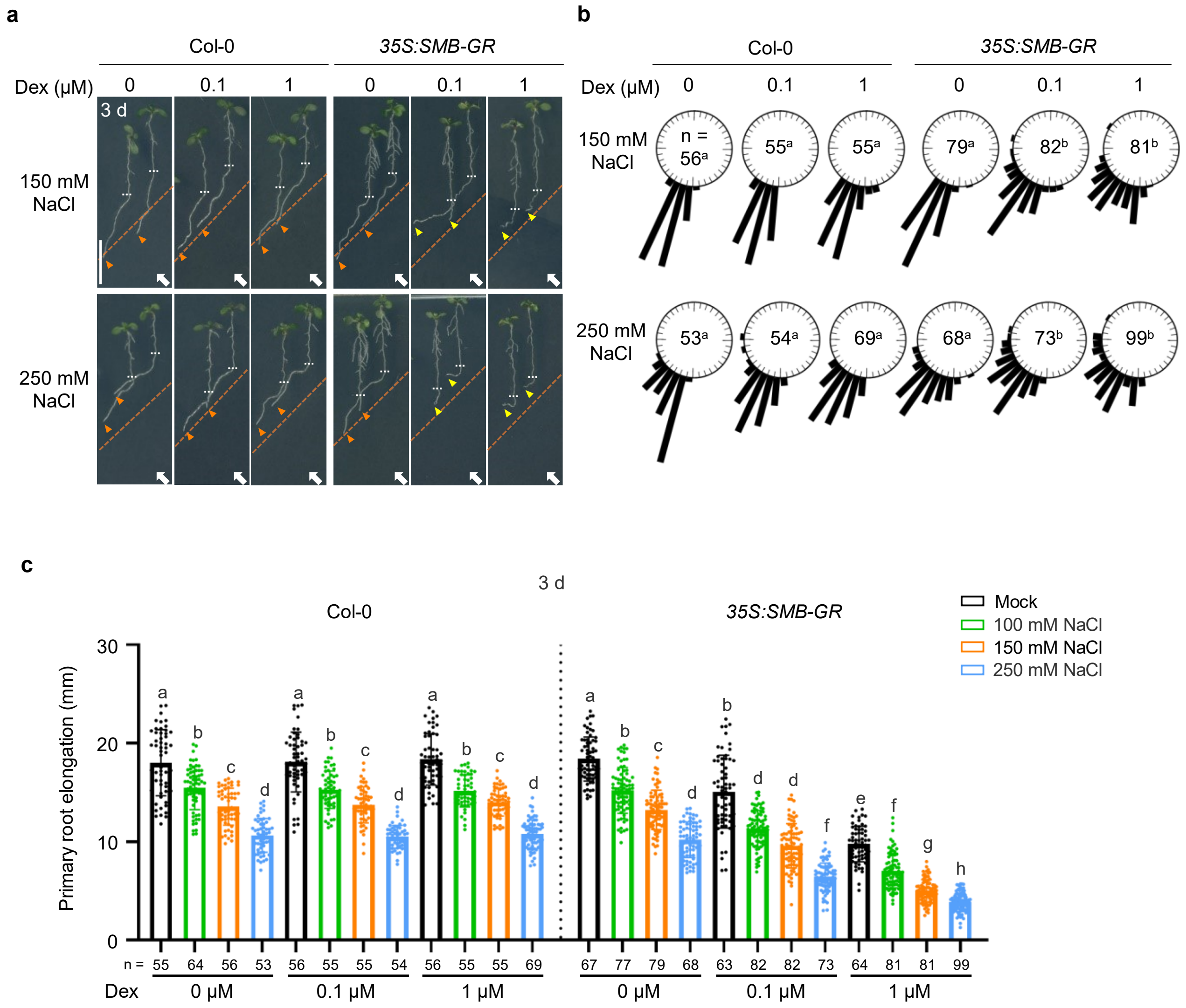

Supplementary Fig. 4. The effects of *SMB* overexpression on halotropic root responses. Related to Fig. 1.

a, b Halotropic root response of Col-0 and *35S:SMB-GR* seedlings that were transferred to split-agar medium containing 150 mM and 250 mM NaCl in the absence or presence of indicated DEX concentrations for 3 days (**a**). White arrows indicate the direction of NaCl diffusion; white dotted lines represent the initial location of the root tip when the salt gradient was created; orange dotted line represents mock-salt boundary. Yellow arrows note enhanced halotropic root response of *35S:SMB-GR* roots upon DEX treatments, compared with Col-0 under corresponding treatments (orange arrows). Scale bar, 1 cm. Halotropic root curvature was quantified and shown in (**b**). **c** Quantification of primary root elongation in Col-0 and *35S:SMB-GR* seedlings that were transferred to split-agar medium containing different concentrations of NaCl in the absence or presence of indicated DEX concentrations for 3

days. Values are means ± SD. In **b** and **c**, n indicates the number of independent seedlings, and alphabets indicate significant differences ($P < 0.05$, two-way ANOVA by Tukey's test).

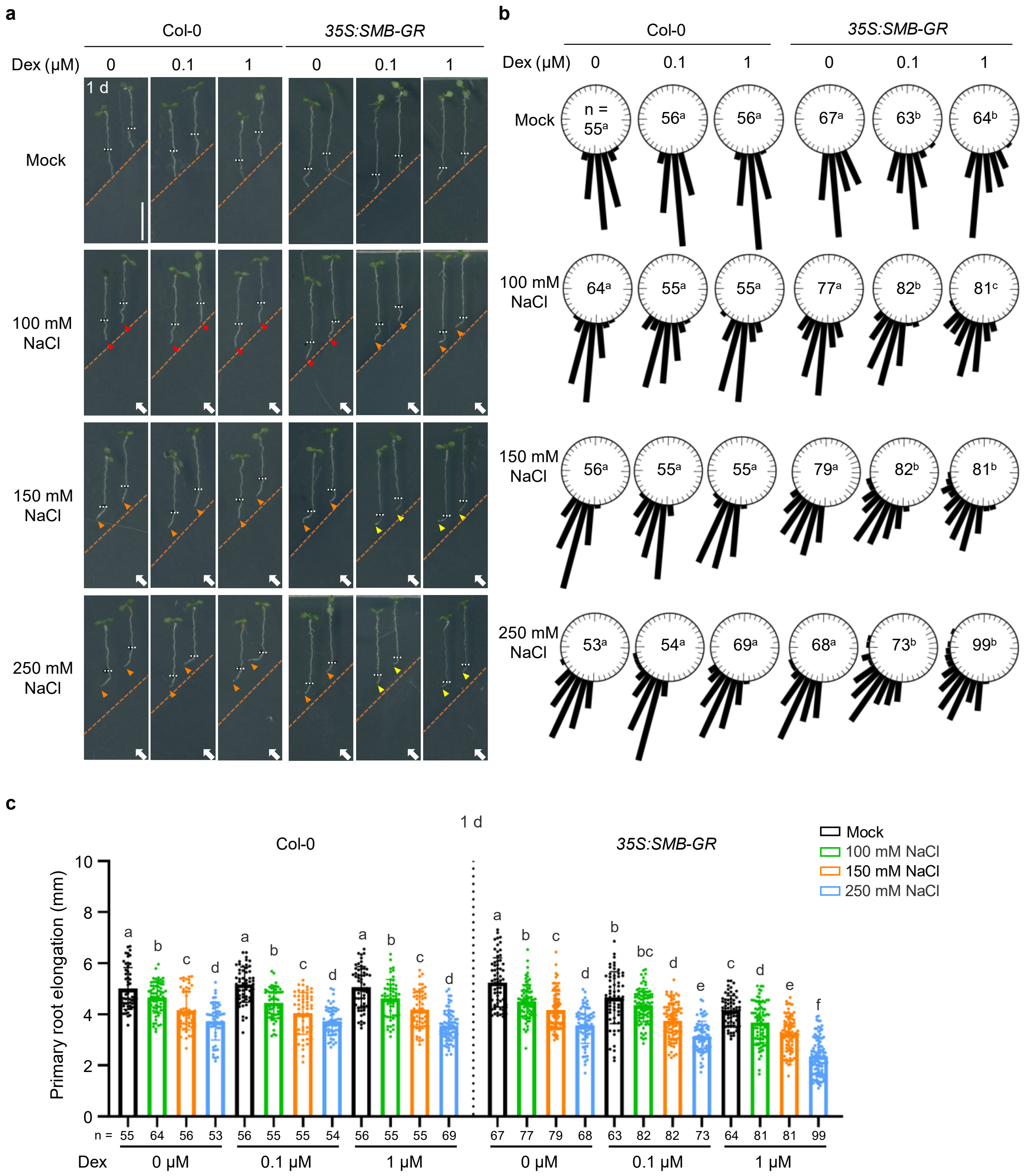

Supplementary Fig. 5. The effects of *SMB* overexpression on halotropic root response for 1d. Related to Fig. 1.

a, **b** Halotropic root response of Col-0 and *35S:SMB-GR* seedlings that were transferred to split-agar medium containing varying NaCl concentrations in the absence or presence of indicated DEX concentrations for 1 day (**a**). White arrows indicate the direction of NaCl diffusion; white dotted lines represent the initial location of the root tip when the salt gradient

was created; orange dotted line represents mock-mock or mock-salt boundary. Yellow arrows note enhanced halotropic root response of *35S:SMB-GR* roots upon DEX treatments, compared with Col-0 under corresponding treatments (orange arrows). Scale bar, 1 cm. Halotropic root curvature was quantified and shown in (**b**). **c** Quantification of primary root elongation in Col-0 and *35S:SMB-GR* seedlings that were transferred to split-agar medium containing different concentrations of NaCl in the absence or presence of indicated DEX concentrations for 1 day. Values are means ± SD. In **b** and **c**, n indicates the number of independent seedlings, and alphabets indicate significant differences ($P < 0.05$, two-way ANOVA by Tukey's test).

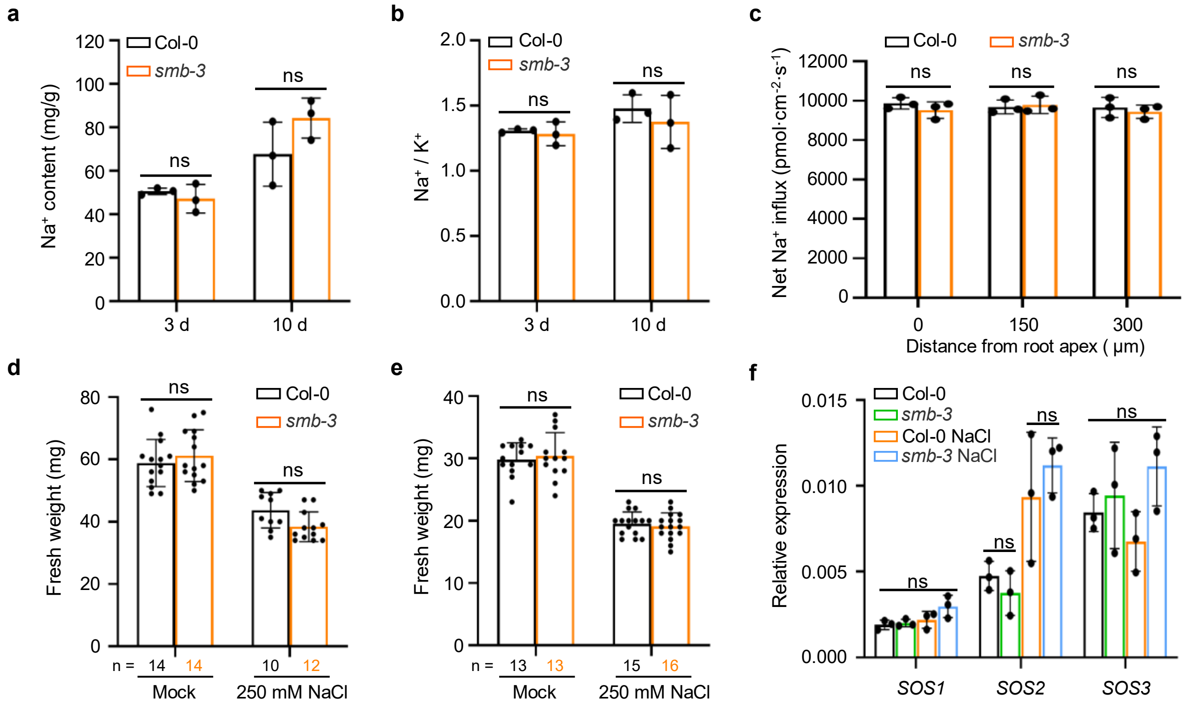

Supplementary Fig. 6. Effects of SMB on endogenous sodium level, plant fresh weight, and salt-tolerance associated gene expression under salt stress. Related to Fig. 1.

a, b Na^+ content and Na^+/K^+ radio of Col-0 and *smb-3* seedlings that were transferred to homogeneous NaCl (100 mM)-containing 1/2 MS plates for 3 and 10 days (n = 3 biological repeats). **c** Quantification of the net Na^+ influx in the root tips of Col-0 and *smb-3* seedlings treated with 100 mM NaCl (n = 3 biological repeats). **d** Quantification of fresh weight of Col-0 and *smb-3* seedlings that were transferred to a split-agar medium with or without 250-mM NaCl for 9 days. **e** Quantification of fresh weight of Col-0 and *smb-3* seedlings that were transferred to medium with or without 100 mM NaCl for 7 days. **f** qPCR analysis of gene expression in the roots of Col-0 and *smb-3* seedlings that were transferred to a split-agar medium with or without 250 mM NaCl for 6 hours (n = 3 biological repeats). Values are means ± SD. In **d** and **e**, n indicates the number of independent seedlings. Statistical analysis was performed with two-tailed Student's *t* test ($P < 0.05$; ns, not significant).

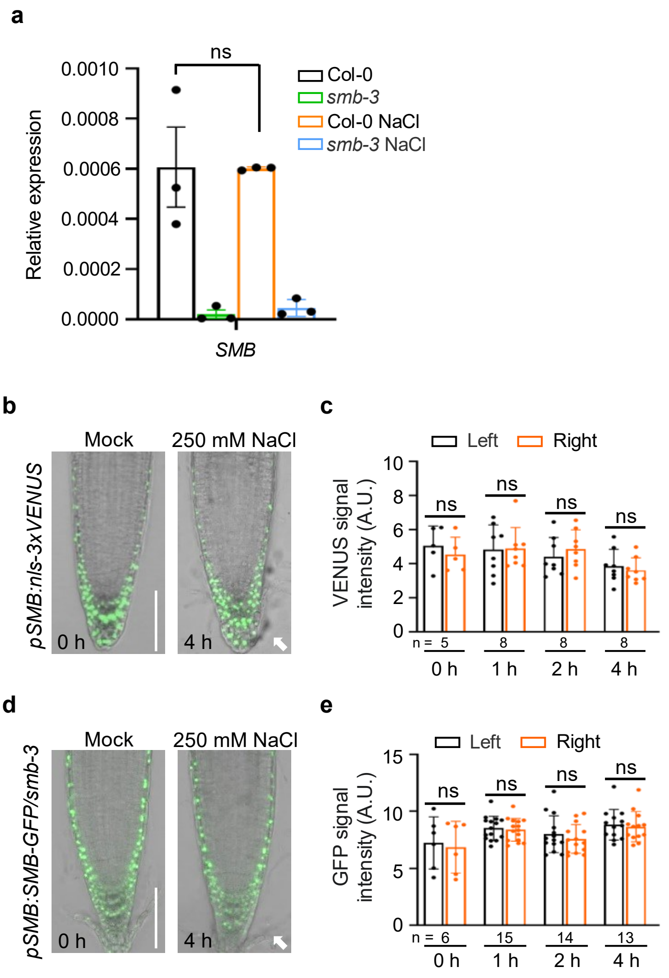

Supplementary Fig. 7. Analysis of *pSMB:nls-GFP* and *pSMB:SMB-GFP/smb-3* expression in root tips at the early stage of halo-stimulation. Related to Fig. 1.

a qRT-PCR analysis of *SMB* transcript in the root of Col-0 and *smb-3* seedlings that were transferred to split-agar medium with or without 250 mM NaCl for 6 hours. Values are means ± SD of three biological replicates. Statistical analysis was performed with two-tailed two-tailed Student's *t* test ($P < 0.05$; ns, not significant). **b**–**e** Expression patterns (**b** and **d**) and quantifications (**c** and **e**) of pSMB:nls-3xVENUS and SMB-GFP in the root tips of respective Col-0 and *smb-3* seedlings that were transferred to split-agar medium with or without 250 mM NaCl over 4 hours. The fluorescence images were overlapped with bright-field images. Similar results were obtained in three independent experiments. Scale bar, 100 μm. The fluorescence intensities of pSMB:nls-3xVENUS (**b**) and SMB-GFP (**d**) in the LRC and epidermis located distal (left) or proximal (right) to mock/salt gradient were quantified. Each point represents an individual data point. Values are means ± SD. In **c** and **e**, n indicates the

number of independent seedlings. Statistical analysis was performed with two-tailed Student's *t* test ($P < 0.05$; ns, not significant).

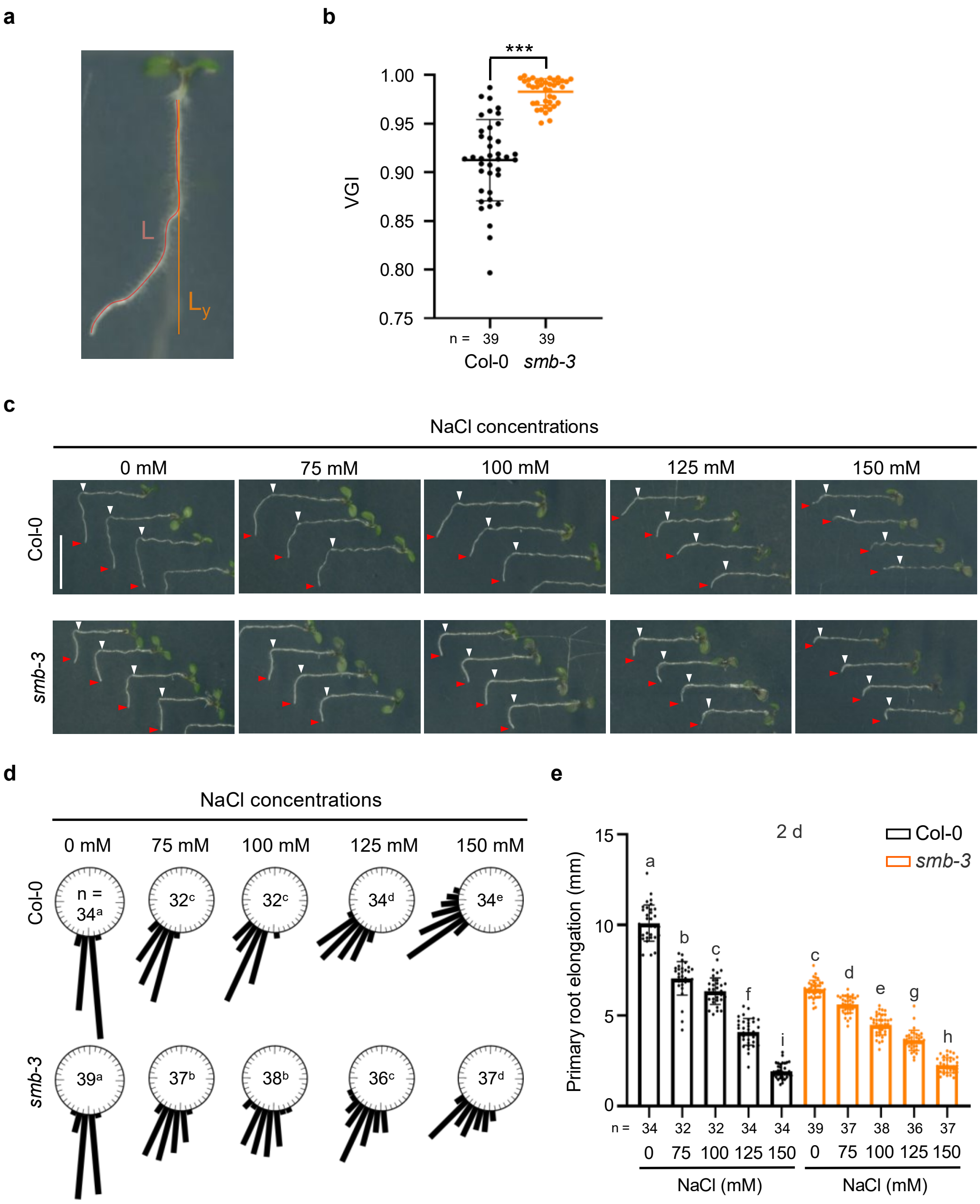

Supplementary Fig. 8. The effect of SMB on gravitropic root growth under different concentrations of NaCl. Related to Fig. 2.

a A schematic diagram defining VGI (See Material and methods). L_y: vertical primary root length (root depth; the orange line); L: total root length (the pink curved line). VGI = L_y/L. **b** Quantification of VGI of Col-0 and *smb-3* seedlings grown in vertical plates after 5 days germination. Values are means ± SD (n = 39 seedlings); statistical analysis was performed with two-tailed Student's *t* test (****P* < 0.001). **c**–**e** Gravitropic root response of Col-0 and *smb-3* seedlings that were transferred to medium with varying concentrations of NaCl and rotated 90 degrees for 2 days gravi-stimulation. The picture was taken 2 days after treatments

(**c**). White arrows represent the position of the root tips when treatment was initiated, and red arrows indicate the position of the root tips after 2 days gravi-stimulation. Scale bar,1 cm. Gravitropic root curvature was quantified and shown in (**d**). 0º equals vertical angle. Primary root elongation was quantified and shown in (**e**). Values are means ± SD. In **b, d** and **e**, n indicates the number of independent seedlings, and alphabets denote significant differences ($P < 0.05$, two-way ANOVA by Tukey's test).

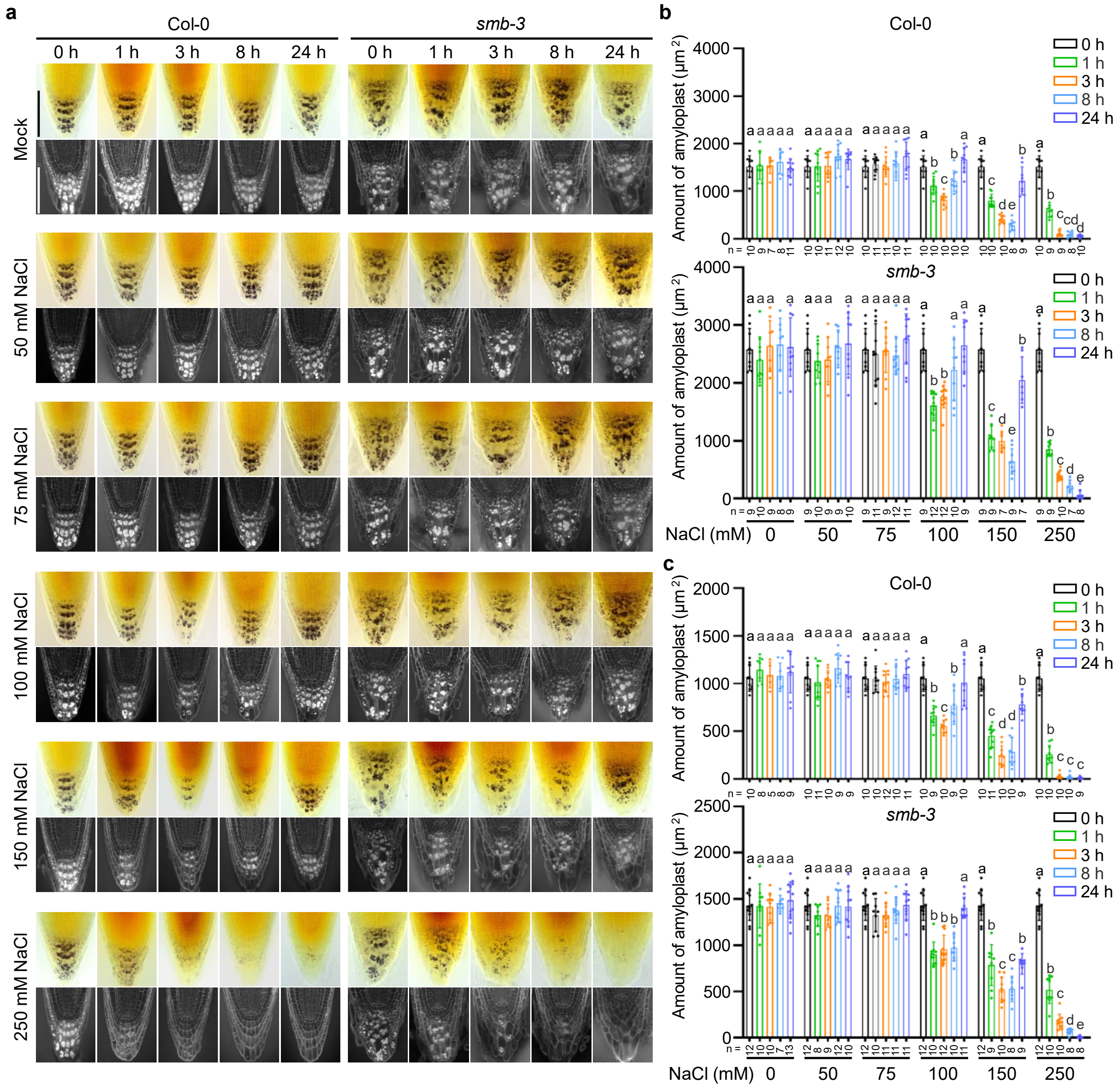

Supplementary Fig. 9. Staining of amyloplasts in the columella cells of Col-0 and *smb-3* under salt stress. Related to Fig. 2.

a–**c** Amyloplasts staining and quantification of the root apexes of Col-0 and *smb-3* seedlings that were transferred to medium containing different concentrations of NaCl at indicated time points (**a**). Similar results were obtained in three independent experiments. Scale bar,100 μm. Amyloplasts area of the root apex was quantified via Lugol's staining (**b**) and mPS-PI (**c**). Values are means ± SD. In **b** and **c**, n indicates the number of independent seedlings, and alphabets denote significant differences ($P < 0.05$, one-way ANOVA by Tukey's test).

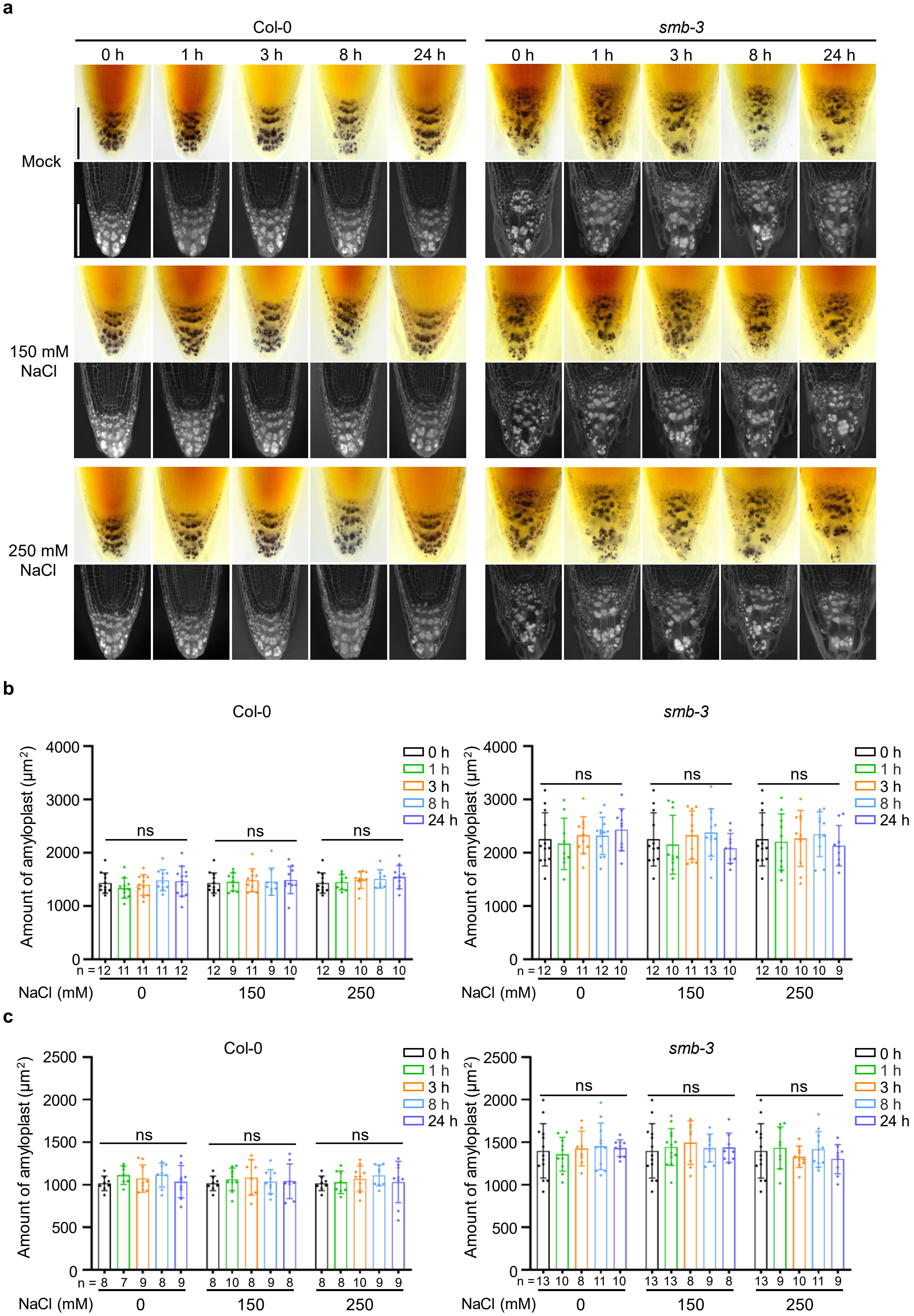
a
Col-0
smb-3
0 h
1 h
3 h
8 h
24 h
Mock
150 mM NaCl
250 mM NaCl
b
Col-0
smb-3
Amount of amyloplast (μm²)
0 h
1 h
3 h
8 h
24 h
ns
n = 12 11 11 11 12 12 9 11 9 10 12 9 10 8 10
n = 12 9 11 12 10 12 10 11 13 10 12 10 10 10 9
NaCl (mM)
0
150
250
c
Col-0
smb-3
Amount of amyloplast (μm²)
n = 8 7 9 8 9 8 10 8 9 8 8 8 10 9 9
n = 13 10 8 11 10 13 13 8 9 8 13 9 10 11 9
NaCl (mM)
0
150
250

Supplementary Fig. 10. Staining of amyloplasts in the columella cells of Col-0 and *smb-3* on halotropic root responses. Related to Fig. 2.

a–c Amyloplasts staining and quantification of the root apexes of Col-0 and *smb-3* seedlings that were transferred to split-agar medium containing different concentrations of NaCl at indicated time points (**a**). Similar results were obtained in three independent experiments. Scale bar,100 μm. Amyloplasts area of the root apex was quantified via Lugol's staining (**b**) and mPS-PI (**c**). Values are means ± SD. In **b** and **c**, n indicates the number of independent seedlings. Statistical analysis was performed with two-tailed Student's *t* test (P < 0.05; ns, not significant).

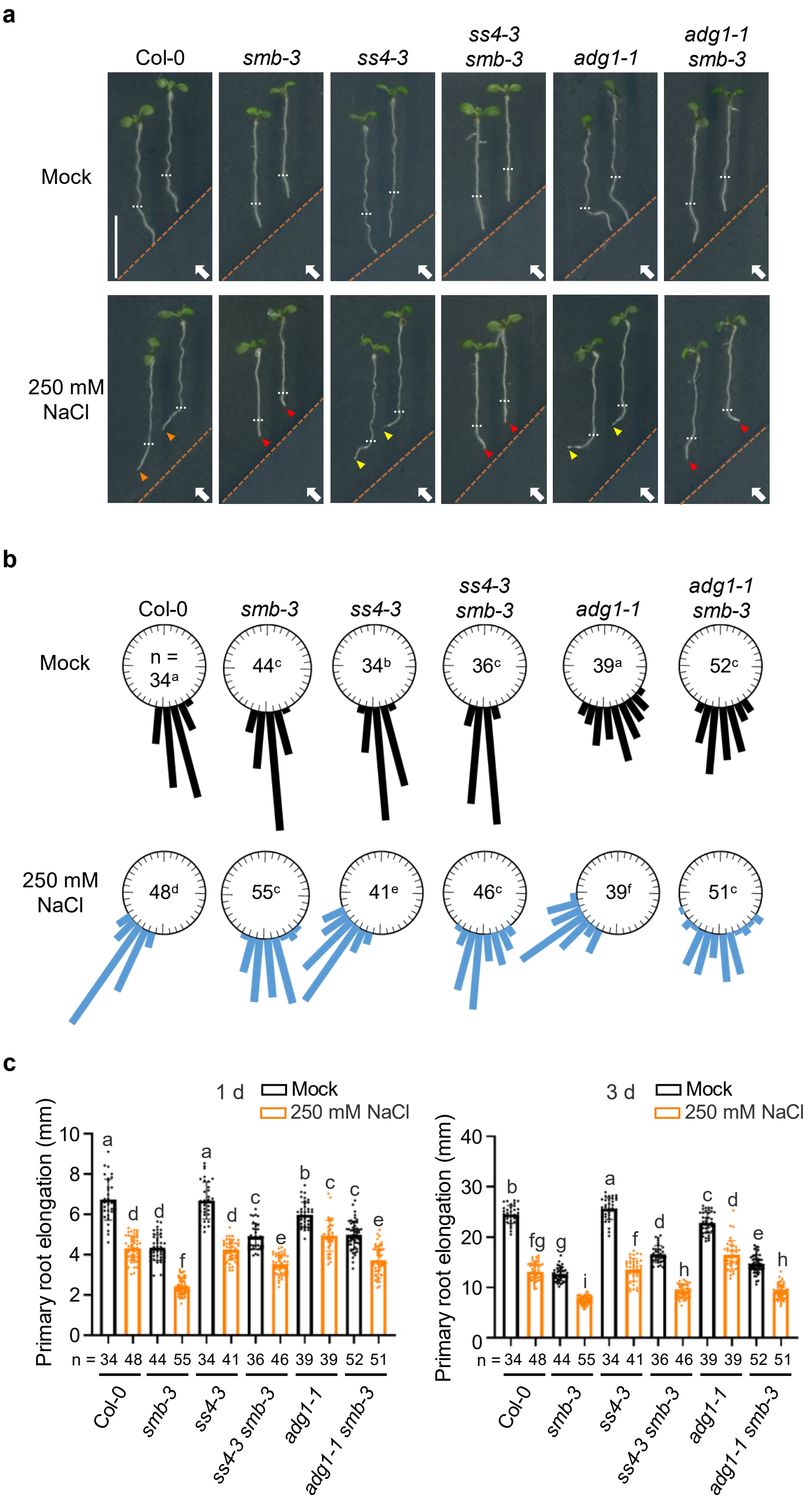

Supplementary Fig. 11. Halotropic root responses of starch synthesis mutants *ss4-3* and *adg1-1* in the regulation of SMB-dependent. Related to Fig. 2.

a, b Halotropic root responses of Col-0, *smb-3*, *ss4-3*, *adg1-1*, *smb-3*, *ss4-3 smb-3* and *adg1-1 smb-3* seedlings that were transferred to split-agar medium with or without 250 mM NaCl

for 1 day (**a**). White arrows indicate the direction of NaCl diffusion; white dotted lines represent the initial location of the root tip when the salt gradient was created; orange dotted line represents mock-mock or mock-salt boundary; orange arrows represent the occurrence of halotropic root bending, while red arrows indicate the absence of halotropic root bending. Yellow arrows indicate accelerated root halotropic bending in the indicated genotypes, relative to that of Col-0 (orange arrows); and red arrows indicate compromised root halotropic response. Scale bar, 1 cm. Halotropic root curvature was quantified and shown in (**b**). **c** Primary root elongation of indicated genotypes were transferred to split-agar medium with or without 250 mM NaCl for 1 day and 3 days. Values are means ± SD. In **b** and **c**, n indicates the number of independent seedlings, and alphabets denote significant differences ($P < 0.05$, two-way ANOVA by Tukey's test).

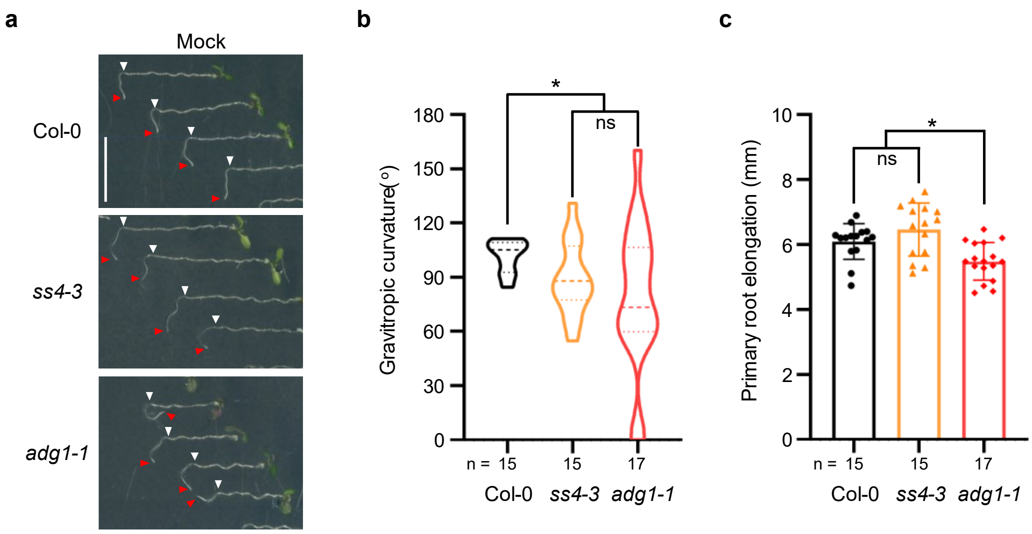

Supplementary Fig. 12. Gravitropic of starch synthesis mutants *ss4-3* and *adg1-1*. Related to Fig. 2.

a–**c** Gravitropic root response of Col-0 and *ss4-3,* and *adg1-1* after 1 day gravi-stimulation. Petri dishes with germinated seedlings were rotated 90 degrees for gravistimulation. White arrows represent the initial position of the root tips when gravistimulation was started, and while red arrows indicate the position of the root tips after 1 day gravi-stimulation (**a**). Scale bar,1 cm. Gravitropic root curvature was quantified and shown in (**b**). Violin plot showing the distribution. Lines represent the upper, median and lower quartile data distribution. Primary root elongation was quantified and shown in (**c**). Values are means ± SD. In **b** and **c**, n indicates the number of independent seedlings. Statistical analysis was performed with two-tailed Student's *t* test (**P* < 0.05; ns, not significant).

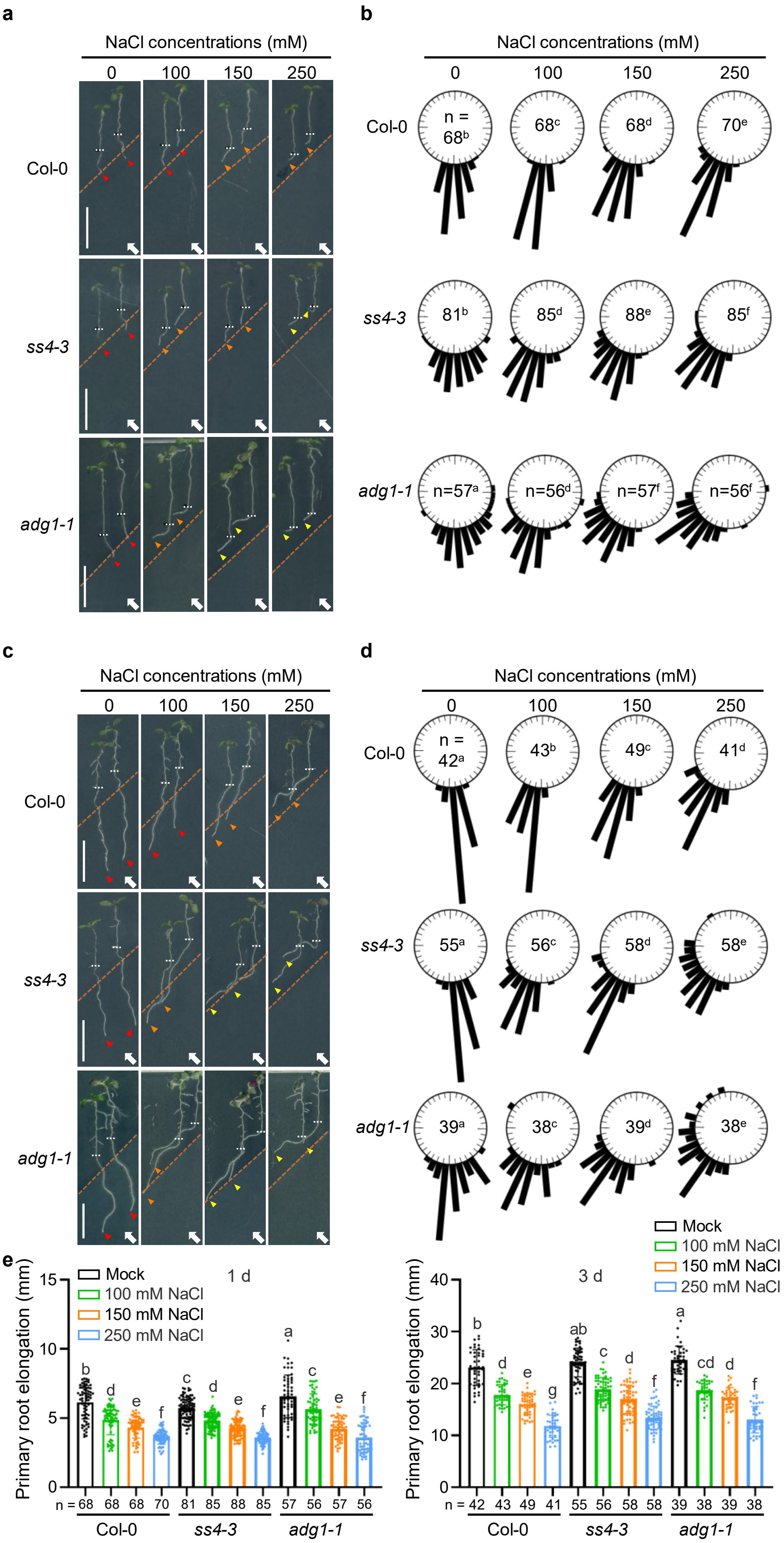
a
NaCl concentrations (mM)
0
100
150
250
Col-0
ss4-3
adg1-1
b
NaCl concentrations (mM)
n = 68b
68c
68d
70e
81b
85d
88e
85f
n=57a
n=56d
n=57f
n=56f
c
d
n = 42a
43b
49c
41d
55a
56c
58d
58e
39a
38c
39d
38e
e
Mock
100 mM NaCl
150 mM NaCl
250 mM NaCl
1 d
3 d
Primary root elongation (mm)
n = 68 68 68 70 81 85 88 85 57 56 57 56
n = 42 43 49 41 55 56 58 58 39 38 39 38

Supplementary Fig. 13. Halotropic root responses of starch synthesis mutants *ss4-3* and *adg1-1*. Related to Fig. 2.

a–**e** Halotropic root response of Col-0, *ss4-3*, and *adg1-1* seedlings that were transferred to split-agar medium containing varying NaCl concentrations for 1 day (**a**) and 3 days (**c**). White arrows indicate the direction of NaCl diffusion; white dotted lines represent the initial location of the root tip when the salt gradient was created; orange dotted line represents mock-mock or mock-salt boundary. Yellow (or orange) arrows note enhanced halotropic root response of indicated genotypes, compared with Col-0 under corresponding treatments (orange arrows), while red arrows indicate the absence of halotropic root bending. Scale bar, 1 cm. Halotropic root curvature was quantified at 1 day (**b**) and 3 days (**d**). Primary root elongation was quantified at 1 day and 3 days (**e**). Values are means ± SD. In **b, d** and **e**, n indicates the number of independent seedlings, and alphabets denote significant differences (P < 0.05, two-way ANOVA by Tukey's test).

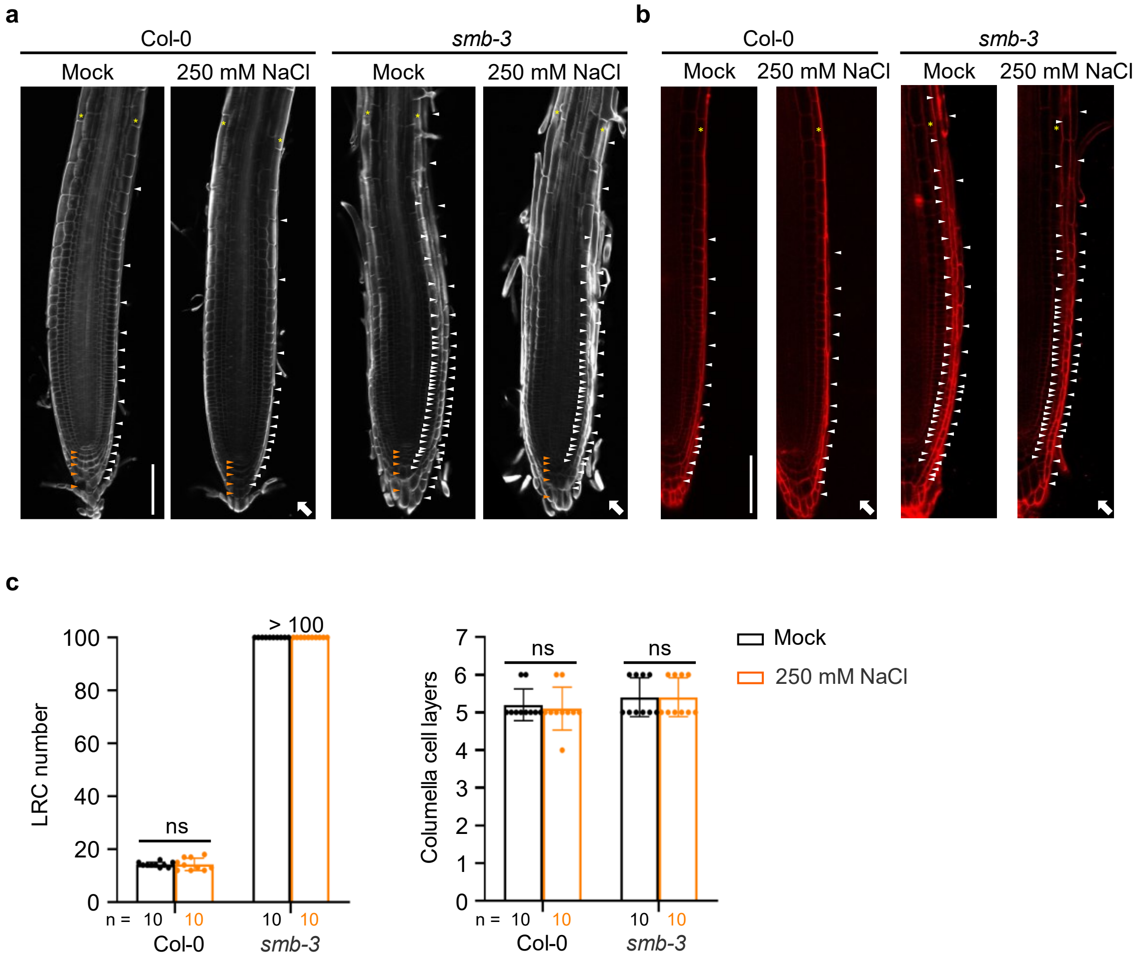

Supplementary Fig. 14. Quantification of lateral root cap cell number and columella cell layer in Col-0 and *smb-3* upon halo-stimulation.

a, b Confocal image of the root apexes of Col-0 and *smb-3* seedlings that were transferred to split-agar medium with or without 250 mM NaCl for 6 hours. The roots of seedlings were cleaned by Clearsee (**a**) or stained with PI (**b**) before imaging. Orange arrow points to the columella cell layer, and each white triangle points to a lateral root cap (LRC) cell. The yellow asterisk represents epidermis cells, and white arrows indicate the direction of NaCl diffusion. Similar results were obtained in three independent experiments. Scale bar,100 μm. **c** Quantification of LRC number and columella cell layers of Col-0 and *smb-3* seedlings that were transferred to split-agar medium with or without 250 mM NaCl for 6 hours. Values are means ± SD; n indicates the number of independent seedlings. Statistical analysis was performed with two-tailed Student's *t* test ($P < 0.05$; ns, not significant).

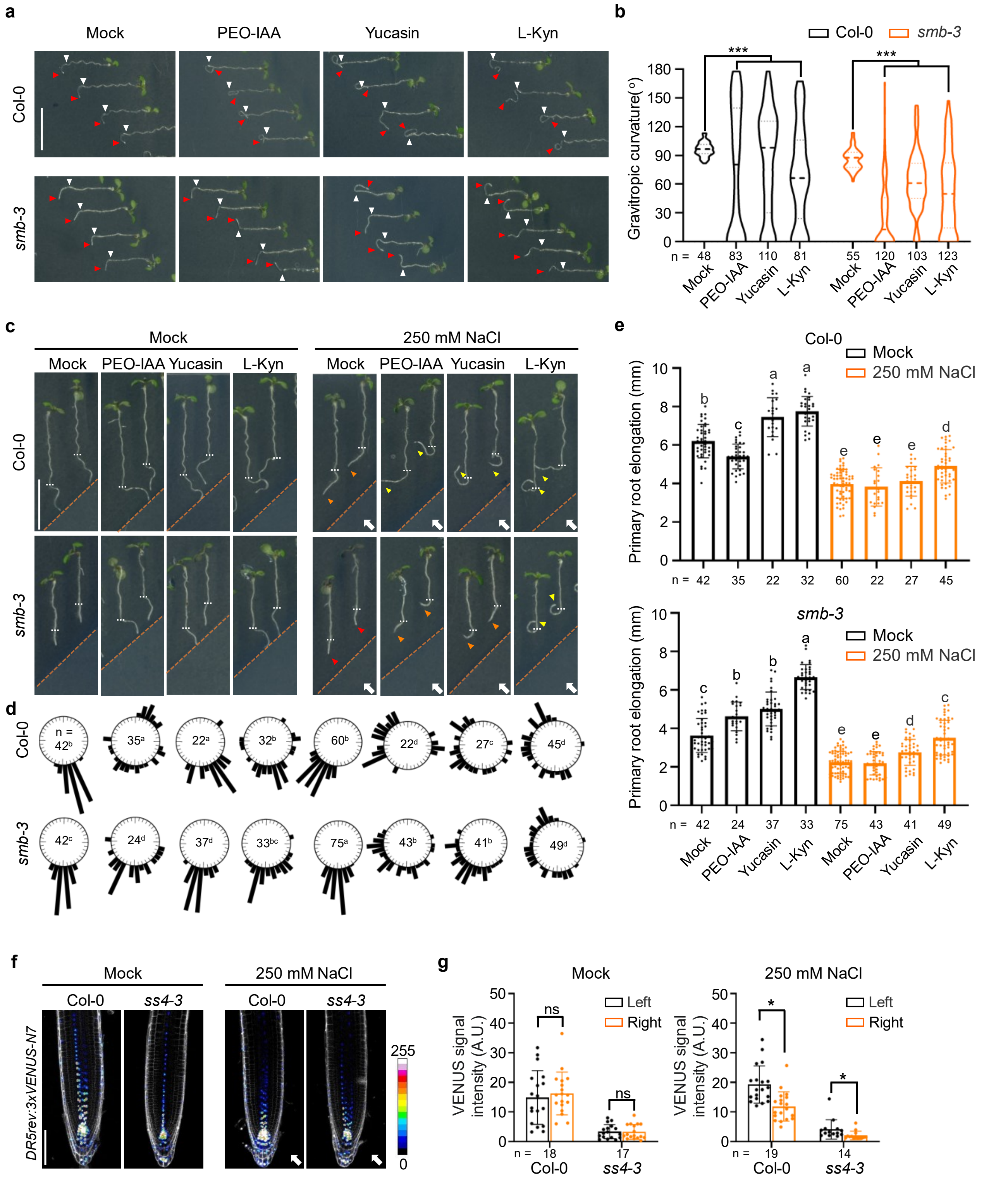

Supplementary Fig. 15. The effects of auxin antagonists on gravitropic and halotropic root responses of Col-0 and *smb-3*. Related to Fig. 3.

a, b Gravitropic root response of Col-0 and *smb-3* seedlings after 1 day gravi-stimulation in the absence or presence of 10 μM PEO-IAA, 10 μM Yucasin or 1 μM L-Kyn. Petri dishes with

germinated seedlings were rotated 90 degrees for gravi-stimulation. White arrows represent the position of the root tips when gravi-stimulation was initiated, and red arrows indicate the position of the root tips after one day gravi-stimulation (**a**). Scale bar,1 cm. Gravitropic root curvature was quantified and shown in (**b**). Violin plot showing the distribution. Lines represent the upper, median and lower quartile data distribution. Statistical analysis was performed with two-tailed Student's *t* test (****P* < 0.001). **c–e** Halotropic root response of Col-0 and *smb-3* seedlings that were transferred to split-agar medium with or without 250 mM NaCl in the absence or presence of 10 μM PEO-IAA, 10 μM Yucasin or 1 μM L-Kyn for 1 day. White arrows indicate the direction of NaCl diffusion; white dotted lines represent the initial location of the root tip when the salt gradient was created; orange dotted line represents mock-mock or mock-salt boundary. Yellow arrows note enhanced halotropic root response of indicated genotypes upon compounds treatments, relative to that of Col-0 (orange arrows); and red arrows indicate the absence of halotropic root bending (**c**). Scale bar, 1 cm. Halotropic root curvature (**d**) and primary root elongation (**e**) were quantified and shown. Values are means ± SD. Alphabets denote significant differences (*P* < 0.05, two-way ANOVA by Tukey's test). **f, g** Confocal images (**f**) and quantification (**g**) of DR5rev:3xVENUS-N7 signal intensity in the root tips of indicated genotypes during 6 hours of halo-stimulation. White arrows indicate the direction of NaCl diffusion (**f**). Similar results were obtained in three independent experiments. Scale bar,100 μm. DR5rev:3xVENUS-N7 signal intensity at the both sides of LRC and epidermal cells was quantified (**g**). Values are means ± SD. In **b**, **d**, **e** and **g**, n indicates the number of independent seedlings. Statistical analysis was performed with two-tailed Student's *t* test (**P* < 0.05; ns, not significant).

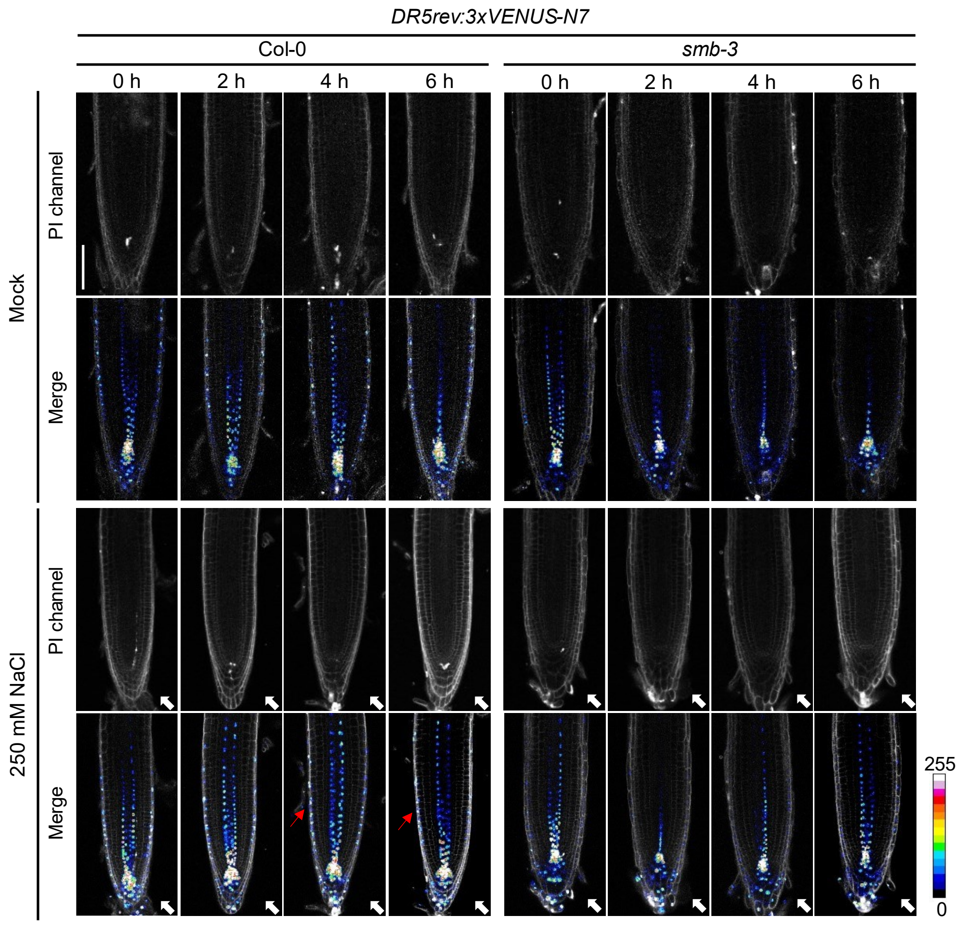

Supplementary Fig. 16. Confocal images of DR5rev:3xVENUS-N7 of Col-0 and *smb-3* upon halo-stimulation over 6 hours. Related to Fig. 3a.

Confocal images of DR5rev:3xVENUS-N7 signal in LRC and epidermal cells of Col-0 and *smb-3* seedlings that were transferred to split-agar medium with or without 250 mM NaCl over 6 hours. White arrows indicate the direction of NaCl diffusion, while red arrows highlight a greater DR5rev:3xVENUS-N7 signal intensity at the distal salt region of root tissues. Similar results were obtained in three independent experiments. Scale bar,100 μm.

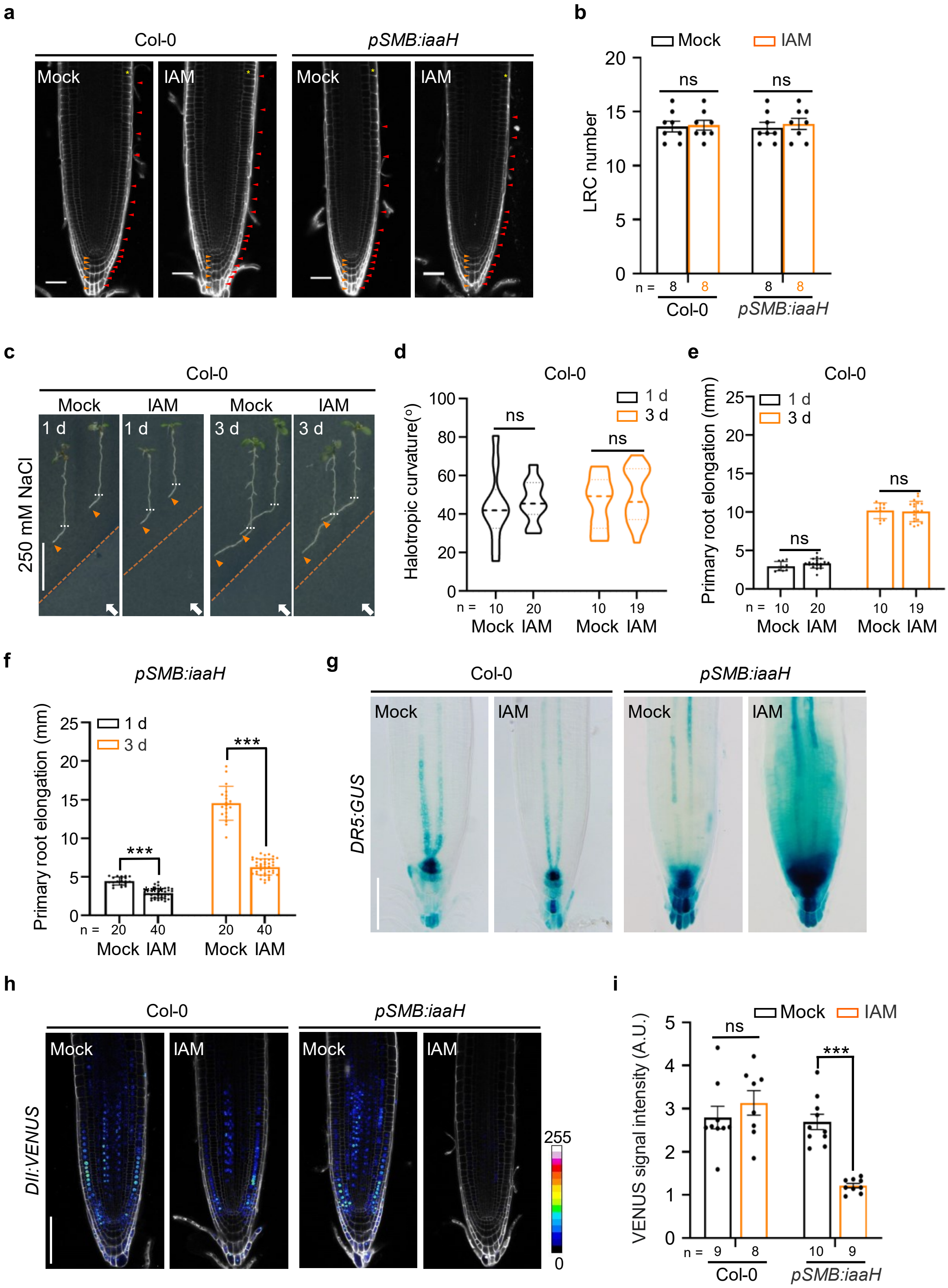

Supplementary Fig. 17. Auxin accumulation in root cap is sufficient to affect auxin accumulation in root tip and halotropic root response. Related to Fig. 3.

a, b Confocal image of the root apexes of Col-0 and *pSMB:iaaH* seedlings that were transferred to medium with or without 1 μM IAM treatment for 6 hours (**a**). Orange arrow points

to the columella cell layer, and each red triangle points to a lateral root cap cell. The yellow asterisk represents epidermis cells (**a**). Similar results were obtained in three independent experiments. Scale bar, 50 μm. LRC number of Col-0 and *pSMB:iaaH* was measured and shown in (**b**). Values are means ± SD. Statistical analysis was performed with two-tailed Student's *t* test ($P < 0.05$; ns, not significant). **c**–**f** Halotropic root response of Col-0 after 24 hours of halo-stimulation in the presence or absence of 1 μM IAM. White arrows indicate the direction of NaCl diffusion; white dotted lines represent the initial location of the root tip when the salt gradient was created; orange dotted line represents mock-salt boundary; orange arrows represent the occurrence of halotropic root bending (**c**). Scale bar, 100 μm. Halotropic root curvature (**d**) and primary root elongation (**e**) was quantified and shown. Violin plot showing the distribution. Lines represent the upper, median and lower quartile data distribution. Values are means ± SD. Statistical analysis was performed with two-tailed Student's *t* test ($P < 0.05$; ns, not significant). **f** Primary root elongation of *pSMB:iaaH* seedlings after 1 day and 3 days of halo-stimulation in the absence or presence of 1μM IAM. Values are means ± SD. Statistical analysis was performed with two-tailed Student's *t* test (***$P < 0.001$; ns, not significant). **g**–**i** Expression patterns of *DR5:GUS* (**g**) and *DII:VENUS* (**h**) in the root tips of Col-0 and *pSMB:iaaH* treated with or without 1 μM IAM for 2 days. Similar results were obtained in three independent experiments. Scale bar, 100 μm. DII:VENUS signal intensities in LRC and epidermis cell were quantified and shown in (**i**). Values are means ± SD. In **b**, **d**, **e**, **f** and **i**, n indicates the number of independent seedlings. Statistical analysis was performed with two-tailed Student's t test (***$P < 0.001$; ns, not significant).

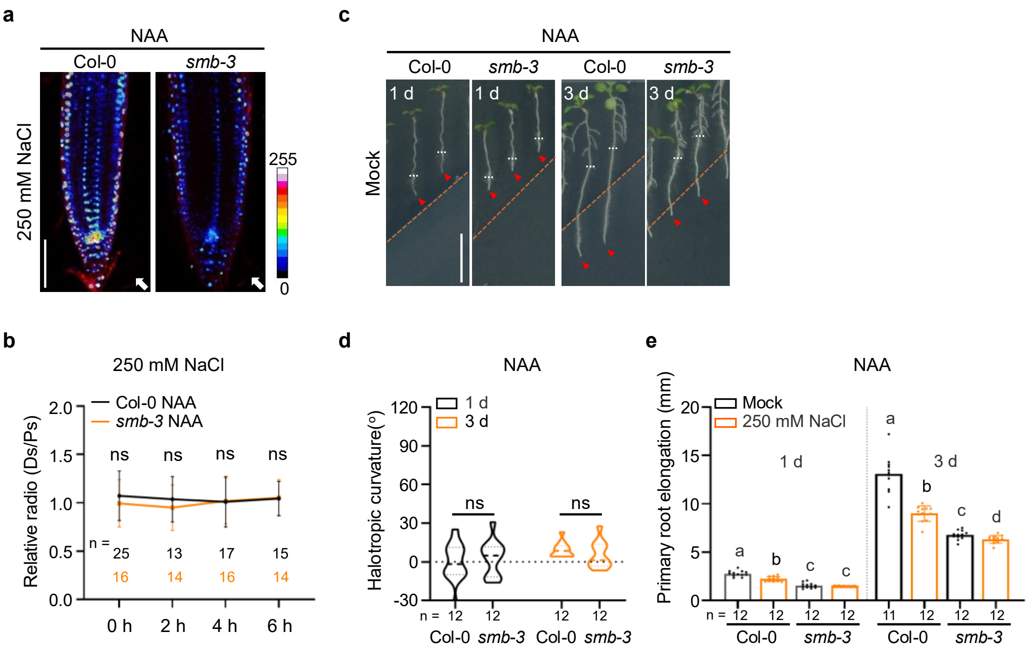

Supplementary Fig. 18. Effects of NAA on auxin accumulation and halotropic root response of Col-0 and *smb-3*. Related to Fig. 3.

a, b Confocal images of DR5rev:3xVENUS-N7 signal in LRC and epidermal cells of Col-0 and *smb-3* after 6 hours of halo-stimulation in the presence of 0.3 μM NAA. White arrows indicate the direction of NaCl diffusion (**a**). Similar results were obtained in three independent experiments. Scale bar, 100 μm. Radio of DR5 signal intensity at distal salt (Ds)/proximal salt (Ps) sides of LRC and epidermal cells over 6 hours was quantified and shown in (**b**). Values are means ± SD. Statistical analysis was performed with two-tailed Student's *t* test ($P < 0.05$; ns, not significant). **c–e** Halotropic root response of Col-0 and *smb-3* seedlings that were transferred to split-agar medium without NaCl in the presence of 0.3 μM NAA for 1 day and 3 days (**c**). White dotted lines represent the initial location of the root tip when the salt gradient was created; orange dotted line represents mock-mock boundary; red arrows indicate the absence of halotropic root bending. Scale bar, 100 μm. Halotropic root curvature (**d**) and primary root elongation (**e**) was quantified and shown. Violin plot showing the distribution. Lines represent the upper, median and lower quartile data distribution. Statistical analysis was performed with two-tailed Student's *t* test ($P < 0.05$; ns, not significant). Values are means ± SD. In **b**, **d** and **e**, n indicates the number of independent seedlings, and alphabets denote significant differences ($P < 0.05$, two-way ANOVA by Tukey's test).

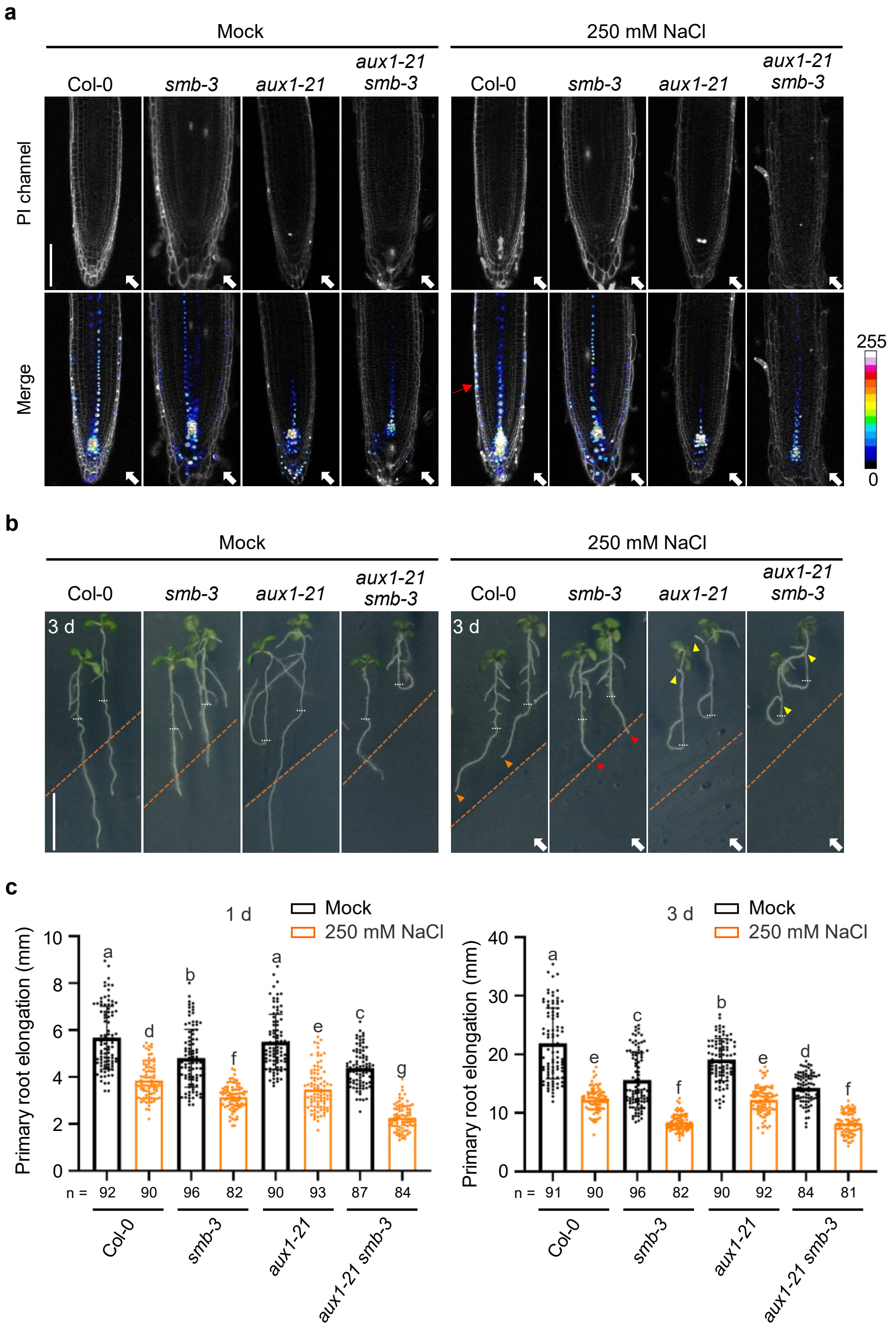

Supplementary Fig. 19. AUX1 mediates the effects of SMB on halotropic root response. Related to Fig. 4.

a Confocal images of DR5rev:3xVENUS-N7 signal in the root tips of Col-0, *aux1-21*, *smb-3*, and *aux1-21 smb-3* seedlings during 6 hours of halo-stimulation. White arrows indicate the direction of NaCl diffusion, while red arrows highlight a greater DR5rev:3xVENUS-N7 signal intensity at the distal salt region of root tissues. Similar results were obtained in three independent experiments. Scale bar,100 μm. **b, c** Halotropic root response of indicated

genotypes after 3 days of halo-stimulation (**b**). White arrows indicate the direction of NaCl diffusion; white dotted lines represent the initial location of the root tip when the salt gradient was created; orange dotted line represents mock-mock or mock-salt boundary. Yellow arrows indicate accelerated root halotropic bending in the indicated genotypes, relative to that of Col-0 (orange arrows); and red arrows indicate compromised root halotropic response. Scale bar, 1 cm. Primary root elongation at 1 day and 3 days of halo-stimulation was quantified and shown in (**c**). Values are means ± SD. n indicates the number of independent seedlings, and alphabets indicate significant differences ($P < 0.05$, two-way ANOVA by Tukey's test).

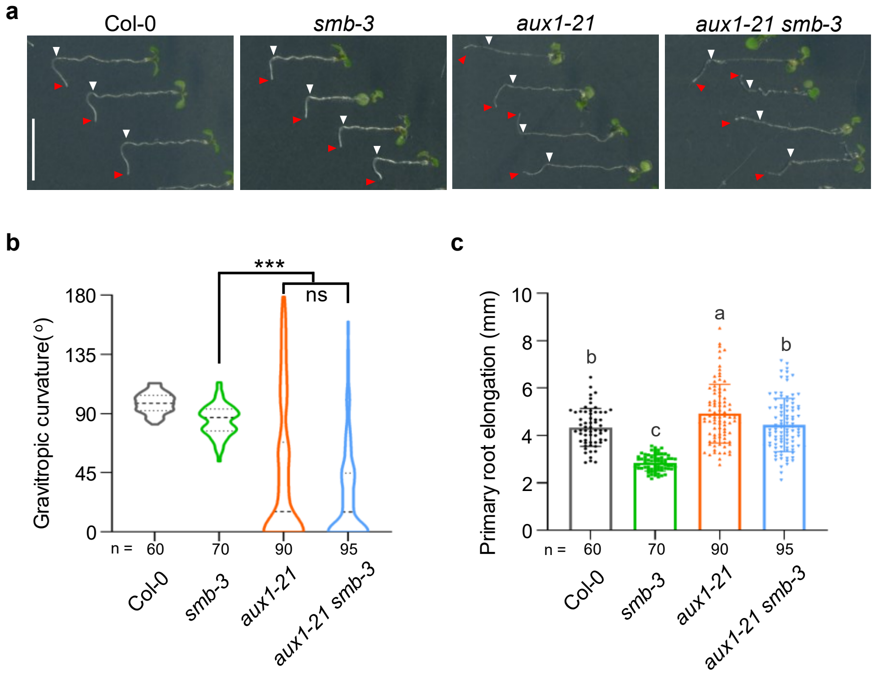

Supplementary Fig. 20. Gravitropic root response of Col-0, *aux1-21*, *smb-3* and *aux1-21 smb-3* upon gravistimulation. Related to Fig. 4.

a–**c** Gravitropic root response of Col-0, *aux1-21*, *smb-3*, and *aux1-21 smb-3* seedlings after one day gravi-stimulation. Petri dishes with germinated seedlings were rotated 90 degrees for gravi-stimulation. White arrows represent the position of the root tips when gravistimulation was initiated, and red arrows indicate the position of the root tips after one day gravi-stimulation (**a**). Scale bar,1 cm. Gravitropic root curvature was quantified and shown in (**b**). Violin plot showing the distribution. Lines represent the upper, median and lower quartile data distribution. Statistical analysis was performed with two-tailed Student's *t* test (***P < 0.001; ns, not significant). Primary root elongation quantified and shown in (**c**). Values are means ± SD. In **b** and **c**, n indicates the number of independent seedlings, and alphabets denote significant differences (P < 0.05, one-way ANOVA by Tukey's test).

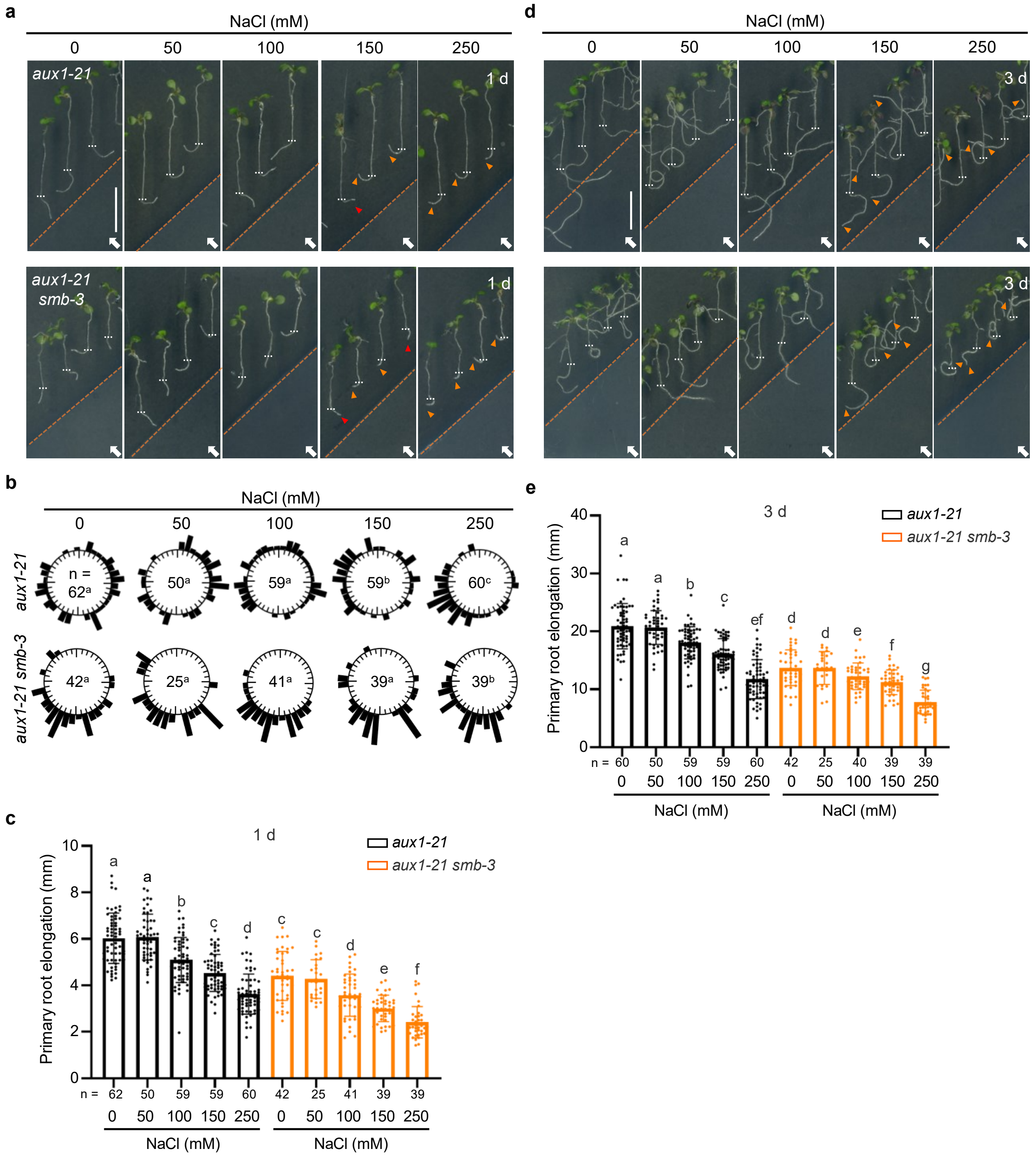

Supplementary Fig. 21. Halotropic root response of *aux1-21* and *aux1-21 smb-3* upon halo-stimulation. Related to Fig. 4.

a–**e** Halotropic root response of *aux1-21* and *aux1-21 smb-3* seedlings that were transferred to split-agar medium containing different concentrations of NaCl for 1 day (**a**) and 3 days (**d**). White arrows indicate the direction of NaCl diffusion; white dotted lines represent the initial location of root tip when the salt gradient was created; orange dotted line represents mock-

mock or mock-salt boundary. Orange arrows represent the occurrence of halotropic root bending, while red arrows indicate the absence of halotropic root bending. Scale bar, 1 cm. Halotropic root curvature was quantified and shown in (**b**). Primary root elongation was quantified at 1 day (**c**) and 3 days (**e**). Values are means ± SD. In **b**, **c** and **e**, n indicates the number of independent seedlings, and alphabets denote significant differences ($P < 0.05$, two-way ANOVA by Tukey's test).

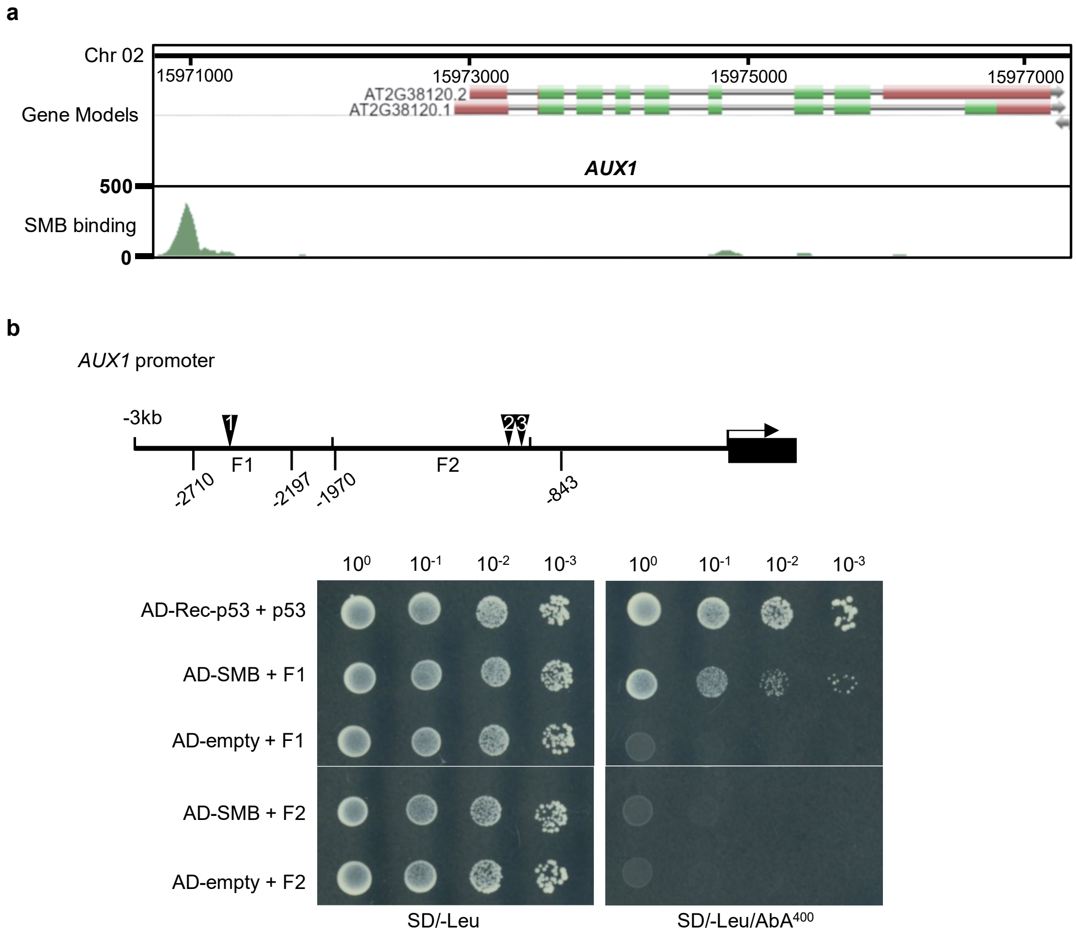

Supplementary Fig. 22. Elucidation of SMB target gene via DAP-seq and yeast one-hybrid (Y1H) experiments. Related to Fig. 4.

a The 2710- to 2197-bp promoter region of *AUX1* was shown as a potential SMB binding site, according to analysis of a released DAP-seq dataset[35]. **b** Validation of putative SMB-binding regions of *AUX1* promoter by the Y1H assay. Yeast cells were co-transformed 0 of 1 with a bait vector containing a promoter fragment F1 or F2 fused to AUR1-C reporter gene, and a prey vector containing SMB fused to a GAL4 activation domain. Yeast cells were grown in liquid medium to an OD60.0 and diluted in a 10× dilution series (10^{-1} to 10^{-3}). From each dilution, 5 µL was spotted onto SD/−Leu medium to select for plasmids, and SD/−Leu supplemented with 400 ng ml^{-1} aureobasidin A (AbA) to select for interaction. p53 was used as a positive. The experiment was repeated three times with similar results.

Supplemental Table 1. Oligos used for genotyping, qRT-PCR, ChIP-PCR, Y1H assay and plasmid construction	
**Name**	**Sequence (5′-3′)**
**Mutant identification**	
LBb1.3	ATTTTGCCGATTTCGGAAC
*smb-3*_F	GTCGTCATCATCATCTGCATC
*smb-3*_R	GTGTATAACGCGCACACAC
*ss4-3*_F	GAATTCAGTGCGTGCGCTAG
*ss4-3*_R	CGCTCCTCAAGTAATGTCACC
*aux1-21*_F	GGCAAAAGCTTCAAAAACCACG
*aux1-21*_R	CTGAATGTTTCACACCTTCCGC
**Expression analysis**	
EF-1α_qPCR_F	CTGGAGGTTTTGAGGCTGGTAT
EF-1α_qPCR_R	CCAAGGGTGAAAGCAAGAAGA
EXPRS_qPCR_F	GGACCTCTGTTGTATCATTTTGCG
EXPRS_qPCR_R	CAACCCTCTTTACATCCTCCAAAC
AUX1_qPCR_F	GGCGATGTACGTGTTGAATG
AUX1_qPCR_R	AACCCTCCACAAACGCATTA
SMB_qPCR_F	GCTTGGACGATAGCGAAAAC
SMB_qPCR_R	TGGTGGTGATGATGATCCAG
SOS1_qPCR_F	CACTTCTGGGAAATGGTTGCA
SOS1_qPCR_R	TGCCTTCAGCAATGACAACAC
SOS2_qPCR_F	CGAAACTTCAAGACAAGGCTC
SOS2_qPCR_R	GTGCCACCTCGTAAATCTCTATC
SOS3_qPCR_F	GTAATGGTGGATAAGGCTTTCG
SOS3_qPCR_R	TGAGCGATGGATTCAAGGATAC
**ChIP-PCR**	
AUX1_ChIP_1_F	AGCAAAAGCAAGCAACTCGT
AUX1_ChIP_1_R	GGCAATGTTGCAGGTCTTCT
AUX1_ChIP_2_F	TGCACATTTCTGGGTTCTTCT
AUX1_ChIP_2_R	TCCGTTTGAGCTGTCACTTG
AUX1_ChIP_3_F	GAATGCTTTCGTGGTGGTTT
AUX1_ChIP_3_R	GGCTGCAGCTGGTTTACATT

**Y1H assay**	
pGADT7_SMB_F	ATACGGGATCCATGGAGATAGGGTCGTCA
pGADT7_SMB_R	TGCAGCTCGAGCTACTTTGGGAACTTGAGAA
pAbAi_AUX1_1_F	GATGAATTGAAAAGCTTGGAGTTGATAATTGAATGCCTA
pAbAi_AUX1_1_R	GAGCACATGCCTCGAGCCTCTCAGTGTTCTCACTAGTCA
pAbAi_AUX1_2_F	GATGAATTGAAAAGCTTTGCCTACCTACCTTTGACCTTAGG
pAbAi_AUX1_2_R	GAGCACATGCCTCGAGCATAACGCCACATGGGTTCA
**Plasmid construction**	
pSMB_iaaH_F	GGGGACAACTTTGTATAGAAAAGTTGGCTCGTTGAAGATGCCTGGATTTA
pSMB_iaaH_R	GGGGACTGCTTTTTTGTACAAACTTGCTATCCTTACTCTTCTTTAAGC
