# Supplementary material for: A root cap-localized NAC transcription factor controls root halotropic response to salt stress in Arabidopsis
Source: Nat Commun. 2024 Mar 7;15:2061. doi: 10.1038/s41467-024-46482-7 (PMC10917740; doi:10.1038/s41467-024-46482-7)
Supplement: Supplementary file 3 — Description of Additional Supplementary Files [file 41467_2024_46482_MOESM3_ESM.pdf]

Description of Additional Supplementary Files:

Supplementary Movie 1: Time-lapse imaging of Col-0 and *smb-3* seedlings after halo-stimulation. Related to Fig.1d. Col-0 and *smb-3* seedlings were transferred to split-agar medium with 250 mM NaCl. Roots were imaged every 5 mins for 72 hours. The obtained pictures were processed by ImageJ software to generate a video. White arrows indicate the direction of NaCl diffusion. Scale bar, 2 mm. Time after the start of halo-stimulation was indicated in the upper left corner.

Supplementary Movie 2: Time-lapse imaging of *DR5rev:VENUS-N7* expression in the root of Col-0 seedling after halo-stimulation. Related to Fig. 3c, d. *DR5rev:VENUS-N7* transgenic seedlings were transferred to split-agar medium with 250 mM NaCl. Roots were imaged every 2 mins for 24 hours. Pink arrows highlight dynamic expression patterns of *DR5rev:VENUS-N7* on the both sides of LRC. White arrows indicate the direction of NaCl diffusion. Scale bar, 0.5 mm. Time after the start of halo-stimulation was indicated in the upper left corner.

Supplementary Movie 3: Time-lapse imaging of *DR5rev:VENUS-N7* expression in the root of *smb-3* seedling after halo-stimulation. Related to Fig. 3c, d. *DR5rev:VENUS-N7/smb-3* seedlings were transferred to a split-agar medium with 250 mM NaCl. Roots were imaged every 2 mins for 24 hours. Pink arrows highlight the dynamic expression patterns of *DR5rev:VENUS-N7* on the both sides of LRC. White arrows indicate the direction of NaCl diffusion. Scale bar, 0.5 mm. Time after the start of halo-stimulation was indicated in the upper left corner.
